# Connections between the Cell Cycle and the DNA Damage Response in Plants

**DOI:** 10.3390/ijms22179558

**Published:** 2021-09-03

**Authors:** Naomie Gentric, Pascal Genschik, Sandra Noir

**Affiliations:** Institut de Biologie Moléculaire des Plantes–CNRS, Université de Strasbourg, 12 Rue du Général Zimmer, 67084 Strasbourg, France; naomie.gentric@etu.unistra.fr (N.G.); pascal.genschik@ibmp-cnrs.unistra.fr (P.G.)

**Keywords:** DNA damage response, plant cell cycle

## Abstract

Due to their sessile lifestyle, plants are especially exposed to various stresses, including genotoxic stress, which results in altered genome integrity. Upon the detection of DNA damage, distinct cellular responses lead to cell cycle arrest and the induction of DNA repair mechanisms. Interestingly, it has been shown that some cell cycle regulators are not only required for meristem activity and plant development but are also key to cope with the occurrence of DNA lesions. In this review, we first summarize some important regulatory steps of the plant cell cycle and present a brief overview of the DNA damage response (DDR) mechanisms. Then, the role played by some cell cycle regulators at the interface between the cell cycle and DNA damage responses is discussed more specifically.

## 1. Introduction

Most multicellular organisms share the same origin, a single fertilized cell. Through the so-called cell cycle, this cell will divide repeatedly to produce the billions of cells constituting the entire organism. A key step of this process is DNA replication, which allows the transmission of two identical sets of genetic information from the mother cell to the daughter cells. In this mechanism, it is of particular importance to avoid the transmission of altered genetic information that potentially could affect the future organism. 

However, living organisms are continuously subject to different types of stress, either endogenous or exogenous, and some of them can compromise the structural or functional integrity of genomes and induce DNA damage. These are referred to as genotoxic stress [1,2,3]. Given the importance of maintaining genomic integrity, cells have developed distinct strategies to detect DNA lesions and respond to genotoxic stress, establishing the DNA damage response (DDR) [1,4].

Even more than other multicellular organisms, in light of their sessile lifestyle, plants are particularly exposed to genotoxic stress and need to integrate developmental and environmental cues to coordinate cell division, cell differentiation, and their postembryonic organ growth. Interestingly, the plant cell cycle machinery relies on an excessive number of cell cycle regulators allowing not only a fine-tuning of the mitotic cycle progression but also a tightly-controlled switch toward the endocycle (or endoreduplication). In this alternative cell cycle, cells duplicate their DNA without cell division, resulting in an increase of their ploidy level. 

While not restricted to plant cells, endoreduplication is widespread in plants, and contributes notably to cell differentiation [5,6]. Lately, it emerged that this whole machinery might confer to plants the cellular plasticity required not only for their development, but also to cope with the occurrence of DNA lesions.

In this review, we briefly describe the regulation of cell cycle focusing on plant particular features to better highlight novel functions in the DNA damage repair of certain key cell cycle players.

## 2. Plant Cell Cycle Progression: Canonical Functions of Some Key Players

**Figure 1:**
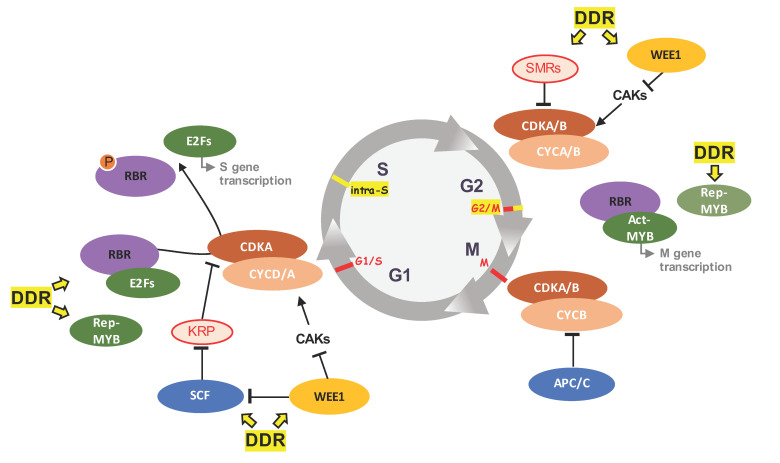
Schematic overview of the plant mitotic cell cycle and its connections with the DDR. Canonical checkpoints, in red, and those activated by the DDR, in yellow, are placed on the cell cycle. Upon detected DNA damage, links where the DDR signalling interfere with cell cycle players are highlighted in yellow. At the G2/M transition, the Rep-MYB TFs are mentioned in light-green as they are degraded by the UPS under normal conditions but accumulate upon DNA damage. Please refer to the core text for further details.

Since the beginning of this century, the fundamental framework of cell cycle progression in plant has been well described (for review [7,8,9]). While the mechanistic basis of the plant cell cycle is comparable to yeast or metazoan, the plethora of cell-cycle-related players, often members of multigene families [10], have already delivered some plant specific features [11], which suggests that others are still pending to be elucidated. As in other eukaryotes, the unidirectional progression of the plant mitotic cell cycle, consisting of the four phases G1 (gap1), S (DNA synthesis), G2 (gap2), and M (mitosis), is driven by the oscillating activities of Ser/Thr protein kinases, i.e., the cyclin-dependent kinase (CDK), whose function and activation are relying on the binding of their cyclin partner (Figure 1).

CDK-cyclin activities increase at both the G1/S and G2/M transitions leading to the phosphorylation of numbers of target proteins resulting in the onset of DNA replication and chromosome segregation, respectively [5,12,13]. Among several classes of CDKs identified in Arabidopsis (i.e., CDK-A to -G and CDK-like kinases) [7,8,10,14], types -A and -B appear directly involved in the core cell cycle machinery. In Arabidopsis, the *Cdk1/2* human homolog, *CDKA;1*, encodes the sole CDK containing a PSTAIRE motif required for cyclin binding. It is constitutively expressed during the cell cycle and likely functions in both S and M phase control [15,16]. Accordingly, its activity is required for entry into S-phase and stem cell maintenance, and this crucial function is based on the control of plant Retinoblastoma homolog (see below [17]). During plant mitosis, A-type CDKs also colocalized with mitotic structures likewise suggesting an important role in the progression of this phase as well [18,19]. 

The plant specific CDKBs are distributed into two subfamilies, CDKB1 and CDKB2, each constituted of two members in Arabidopsis, and characterized by the PPTALRE and PS/PTTLRE cyclin-binding motifs, respectively [10,20]. A remarkable feature of these *CDKB* genes is their transcriptional cell cycle-dependent regulation, in which *CDKB1s* accumulate during the S phase until the G2 phase, and *CDKB2s* are more restricted to the G2 and M phases [14,20], insuring a maximum of kinase activity at the G2-M transition and during early mitosis, respectively [21]. In term of function, CDKB2s are required for the maintenance of normal mitotic activity and for both shoot and root meristem organization and development [22]. Regarding Arabidopsis, it was reported that in combination with CYCA2;3, the B1-type CDKB1;1 acts as a mitotic activator and a negative regulator of endocycle onset [23,24]. More recently, it was shown that CDKB1s, by enabling to maintain cell cycle progression even in Arabidopsis *cdka;1* null mutant, might exhibit partially redundant function with CDKA;1 and allow finetuning of the cell cycle destiny [17,25].

As mentioned above, an essential step in CDK activation involves their periodic association with a regulatory cyclin partner whose oscillating levels are precisely controlled by transcription and protein stability (see reference below and [26]). With more than 40 cyclins in Arabidopsis, plants present multiple members of the core cyclins commonly described in higher eukaryotes and distributed, with some exceptions, into the three main groups: D-type (G1-S transition), A-type (S to M progression), and B-type (mitotic) cyclins [14,21]. 

Globally, CYCDs interact with CDKA;1, while CYCAs and CYCBs interact with both A- and B-type CDKs [21,27,28]. Nevertheless, given the multiple members of each cyclin class, only few CDK-binding partner and biological significance have been identified in higher plants thus far [21,29,30,31]. Interestingly, while in yeast, cyclins have been suggested to provide the substrate specificity of the CDK-cyclin complexes [32,33], in plants, this specificity appears to be determined by the accurate matching of both the CDK and the cyclin [17].

Moreover, the full activation of CDKs requires not only cyclin binding but also CDK phosphorylation. This positive phosphorylation event occurs at a canonical threonine residue within the T-loop of CDK proteins and is mediated by CDK-activating kinases (CAKs) [34,35,36]. In Arabidopsis, these kinases correspond to two gene classes, the *D-* and *F*-type *CDK*s, with three and one members, respectively [34,37,38]. CDKF;1 appears plant-specific and functions as a CAK-activator of Arabidopsis CDKDs [34,37]. Among them, CDKD;1 and CDKD;3 in association with CYCH, phosphorylate and activate all core CDKs (CDKA, CDKB1, and CDKB2) [39,40], triggering plant cell cycle mitotic activity [38].

Interestingly, inhibitory phosphorylation of CDKs (at tyrosine and threonine residues within the so-called P-loop region) constitutes a major mechanism in metazoan cell cycle regulation and is mediated by the Wee1 kinases. As a counterbalance, the Cdc25 phosphatases release the CDKs from the inhibitory phosphate group enhancing the cell cycle progression towards both M and S phases (for review [41]). This on-off switch of CDK activities upon control of the Wee1/Cdc25 module appears a key feature in fungi and metazoans to insure the unidirectionality of the cell cycle, which also contributes to cell cycle arrest in case of DNA replication stress. Despite the identification of structural plant homologs [42,43], the control in M phase entry or the orderly cell cycle progression does not rely on a WEE1/CDC25 module in plants, and the roles of both Arabidopsis WEE1 and, to an even less extent, the putative CDC25, are not yet clearly established under normal plant growth conditions. Indeed, in Arabidopsis, the WEE1 kinase downregulates CDKD activity by Tyr23/24 phosphorylation [40] and, is also able to phosphorylate CDKA;1 (Thr14, Tyr15) [44]; however, this phosphorylation does not affect the CDKA;1 activity level [45]. Those data highlight a major evolution dissimilarity between plant and metazoan cell cycle regulation [11,46].

CDK activity decay being required to enable progression towards mitotic exit and further G1 entry in order to license replication origin required for a new DNA synthesis round [7], substrate phosphorylation activity of the CDK-cyclin complexes is also countered by direct binding with so-called CDK inhibitors (CKIs). Plant genomes encode noticeably more CKIs than metazoan or fungal genomes, and distinguish two families, the KIP-RELATED PROTEIN/INTERACTOR OF CDKs (KRP/ICK) [47,48], and the plant specific SIAMESE/SIAMESE-RELATED (SIM/SMR) [49,50], with, respectively, 7 and 17 members in the Arabidopsis genome. 

KRPs play, in a dose-dependent manner, an essential role in the canonical cell cycle regulation and the setting of the G1 checkpoint. At low levels of expression, KRPs repress mitotic activity and promote endoreduplication, while, at high expression levels, they will block mitosis and DNA replication [51,52,53]. The SMR proteins appear inactive during the G1 phase and the G1/S transition, enabling S phase progression, and then, in mitotically arrested or endocycling cells, SMRs maintain the blocking into mitosis entry, contributing to the establishment of a G2 checkpoint. This large family of CKI genes appears to act as integrators of environmental and developmental signals to fine-tune cell cycle regulation in plants [50,54,55].

Orchestrated cell cycle events are also maintained by the oscillatory transcription of phase-specific genes [56,57]. In the plant field, those regulations depend on one hand, on the control of a set of early cell cycle (G1) genes by the canonical E2F transcription factors (TF) combined to their dimerization partner (DP), and on the other hand, on the regulation by the MYB3R TF family of a set of late cell cycle (G2) genes, containing M-phase specific activator (MSA) *cis*-elements (for reviews [12,58]). Those TF activities are themselves controlled by their sequential interactions with the multifunctional master regulator, known as RETINOBLASTOMA-RELATED 1 (RBR1) in Arabidopsis [59,60]. 

The evolutionary conserved Retinoblastoma (Rb) protein, initially described in human as a tumour suppressor, negatively regulates the cell cycle progression through its interaction with the heterodimer E2F-DP [61]. In Arabidopsis, it is yet clearly established that RBR1 function is not restricted to the sole control of cell proliferation [62]. Based on its phosphorylation status, controlled at least by the CDKA;1 [17] and S6K [63] kinases, and depending on the developmental stage of a given tissue (i.e., meristematic or differentiated), RBR1 will take part in the RBR1-E2F complex and/or in a multiprotein complex, the so called DREAM-like complex [60,64]. Indeed, initially described in nematode, drosophila, and mammalian cells, the multiprotein DRM/dREAM/DREAM complex (DP, Rb-like, E2F, and MuvB) constitutes a key regulatory machinery ensuring cellular quiescence by repressing cell cycle regulators and coordinating phase-specific gene expression (for review [61]). In Arabidopsis, RBR1 together with E2FB and E2FC (but not E2FA thus far) and their DP partners, as well as MYB3R factors might also constitute such a complex to integrate plant internal and environmental cues and repress or activate the cell cycle machinery [59,65].

Finally, throughout the whole plant cell cycle, its non-reversible progression appears to rely, as for yeast, fungus and metazoan, on the selective and temporally-regulated degradation of key cellular players by the ubiquitin-proteasome system (UPS; reviewed in [66]). In particular, the CULLIN-RING (CRL)- and monomeric-RING- types of E3 ubiquitin-ligases might play a dominant role in the regulation of CKI protein levels involved in the G1/S transition and/or the shift towards the endocycle [55,67,68,69,70]. Likewise, the plant ANAPHASE PROMOTING COMPLEX/CYCLOSOME (APC/C) E3 complex [66,71] by targeting, as in animals, mitotic CYCs exhibiting a D-box motif, and through the activity of co-activators CCS52A1/A2 (CELL CYCLE SWITHCH52), controls at least both mitosis progression and endoreplication onset [72]. At present, the limited number of identified E3 ubiquitin ligases together with their substrate repertoire offer a wide exploratory research field.

## 3. The Plant DDR Machinery

Concomitantly with the highly controlled cell cycle progression, cells have to deal with DNA damage occurring through endogenous stress, such as DNA replication and recombination, or ROS metabolism, as well as abiotic and biotic stresses. The DDR relies on a complex signalling network, composed of different layers of players, i.e., the sensor, mediator, transductor, and effector proteins. They specifically detect and respond to DNA lesions, in order to prevent the transfer of erroneous genetic information and to not compromise the fitness of the organism it belongs to (reviewed in [3,73,74,75]).

During evolution, plants and animals have partly conserved the DDR machinery and both present two distinct pathways regarding the kind of DNA damage [76]. The Ataxia Telangiectasia Mutated (ATM) pathway is activated by the MRE11/RAD50/NBS1 (MRN) sensor complex in response to DNA double strand break (DSB) and the ATM- and RAD3-related (ATR) pathway, which is activated by single-stranded DNA damage, detected by different sensors, such as the RPA complex (reviewed in [77]) or the 9-1-1 complex [78]. 

The two Ser/Thr kinases ATM and ATR constitute the main transductors of the DDR signal and modulate effector protein activity through their phosphorylation action. In animal, this DNA damage signal can be either direct or relayed by Chk2 and Chk1, two other checkpoint kinases absent in plants [74], to activate the central effector, the tumour suppressor protein p53, a transcription factor able to control around hundreds of genes in response to DNA damage [79]. Plants also lack p53; however, the characterization of DDR in Arabidopsis allowed identification of the NAC (NAM, ATAF1/2, CUC2) transcription factor SOG1 (SUPPRESSOR OF GAMMA RESPONSE 1) as the functional homolog of p53 [80,81]. Thus, SOG1 was shown to be directly phosphorylated by ATM on five serine residues (SQ350, SQ356, SQ372, SQ430, and SQ436), allowing the increase of SOG1 affinity for its target gene promotors [82,83]. Moreover, by in vitro assay, it was shown that SOG1 is also phosphorylated by ATR, resulting in a different transcriptional response than the one induced by ATM [84]. Recently, a functional and temporal transcriptome analysis revealed that SOG1 is a master regulator of the DNA damage response. The expression of more than a thousand of genes is SOG1-dependant, though those genes can be or not direct targets of the TF, and interestingly, while this is not the case of p53, Gene Ontology analysis highlighted the particular implication of SOG1 in targeting numerous genes required for DNA repair by homologous recombination (HR) [85,86].

Based on the type of the detected DNA damage, distinct cellular responses can be established. The first response to be favoured is the cell cycle arrest allowing the set-up of appropriated DNA repair mechanisms. This response is driven by the activation of cell cycle checkpoints, notably upon the control of WEE1 and SMR regulators [87] and the transcriptional inhibition of factors required for cell cycle progression, such as *CDKB2;1* and *KNOLLE* [81]. 

Notably, the repertoire of DNA repair mechanisms is as extensive as the repertoire of DNA errors or lesions and, thus, allows specific responses [1,87,88,89]. Briefly, these include the nucleotide and base excision repair pathways (NER and BER), the mismatch repair pathway (MMR), and two pathways involved in DNA DSB repair, the Non-Homologous-End-Joining (NHEJ), and the Homologous Recombination (HR), also called homology-dependent repair.

DSBs, which can result in the loss of a DNA fragment up to entire genes, constitute the most critical DNA damage [90]. Once localised at the DSB sites, the MRN complex recruits and activates the ATM kinase, further activating SOG1 and the SOG1-dependent responses. Simultaneously, both ATM and ATR are able to target the histone variant H2AX to induce its phosphorylation (γ-H2AX) in euchromatin [91,92], and the accumulation of γ-H2AX at the DSB sites acts as a signal for the recruitment of DDR proteins [93]. Interestingly, ATM is also able to target the plant-specific histone variants H2A.W.7, a marker of DSB at the heterochromatin level to induce its phosphorylation [94]. 

In the NHEJ repair mechanism, active throughout the cell cycle, though particularly active during the G1 phase [95], DNA ends are joined with no respect of the original sequence, resulting in a loss of genetic information [96]. Conversely, HR takes place in the S/G2 phase as it requires the presence of a sister chromatid used as a template to allow repair with minimal errors [97]. Currently in plants, it is postulated that, to repair DNA DSBs, meiotic and mitotic cells would favour the error-free HR pathway, while the majority of DSBs in somatic cells, most of them being differentiated, would be repaired via the NHEJ pathway [98,99].

Finally, if the DNA damage is too severe to be repaired, two scenarios permit the cell to prevent the transmission of incorrect genetic information. Either the cell operates a switch toward programmed cell death (such a fate appears particularly true in meristematic tissue around stem cells [100,101]), or the cell drives a transition of the mitotic cycle towards endoreduplication [102,103]. Establishing a differentiated cell status is a common strategy in plants and will be further discussed below.

## 4. Beyond Cell Cycle Regulation: Cell Cycle Players in DDR

In this section, we highlight some players or mechanisms in plants acting at the interface between cell cycle regulation under normal growing conditions and DNA-damage/repair mechanisms upon DNA-lesion-inducing stresses (Figure 1).

### 4.1. Functional Specialization of CDK/CYC Complex in DDR

Detection of DSB-type of lesions engages cells to decide whether to perform DNA repair by HR or NHEJ pathway. While the inhibition of Cdk activity is required upon DNA damage to block cell division, clear evidences in yeast and mammals show that Cdk activity is also critical for the coordination and the execution of DNA-end resection during HR and, thus, the activation of the checkpoint response [104]. In plants, such a dilemma appears to be solved by the functional specialization of the CDK/CYC complex. 

**Figure 2:**
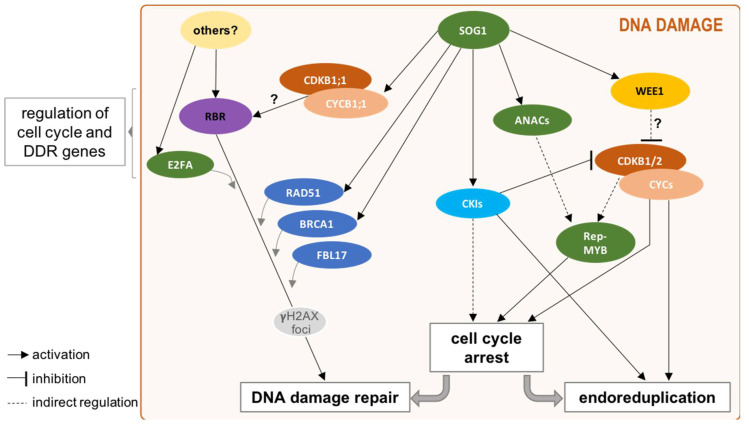
The SOG1-independent DDR signalling appears to favour the onset of DNA damage repair mechanisms. RBR, together with E2FA are key regulators of both cell cycle and DDR genes in the absence of DNA lesions [62]. Upon DNA damage, the SOG1 transcription factor overtakes the transcriptional regulation response to fine-tune DDR signalling. Thus, among hundreds of targets, the WEE1 kinase, other ANAC transcription factors or CKI proteins, such as *SMR4*, *SMR5*, *SMR7,* and *KRP6,* are activated. By inhibiting the CDK/CYC complex, other regulators, such as the Rep-MYBs, are not further phosphorylated, and these TFs accumulate in the cell, hence, contributing to block the cell cycle progression. Then, to avoid the transmission of damaged DNA, the cell will either switch towards endoreduplication or proceed to DNA lesion repair. In this option, the RBR-E2F pathway can be activated to setup DNA repair mainly by homologous recombination, this signalling pathway could be activated either via the activity of the CDKB1;1/CYCB1;1 complex, or via a SOG1-independent activation from upstream components not yet identified. Finally, if none of these alternatives is reached, then cell death will occur. A question mark (?) indicates that further experimental data are needed for confirmation of the indicated connections.

In particular, within the plant specific B-type CDKs, CDKB2s described as mitotic regulators in Arabidopsis, are actually transcriptionally repressed by SOG1 upon DNA damage [22,81], while CDKB1s, only when combined with the B1-type CYCs, constitute major regulators of plant HR [99] (Figure 2). Upon DNA damage, CYCB1;1 (and not CYCB1;2) is targeted by SOG1, here reflecting the functional specialization in DNA repair of the CYCB1;1 [105], thus, promoting CDKB1 activity and the HR execution [81,99].

### 4.2. DREAMs upon DNA Damage

As previously mentioned, upon DNA damage, SOG1 is the major transcriptional activator directly targeting genes the most strongly induced in such conditions [80,81,86]. Namely, by activating inhibitors of the cell cycle progression, such as the CKI proteins and WEE1 kinase, its implication in cell cycle arrest was clearly established [44,85,86] (Figure 2). Notably, during cell cycle progression, it is described that once phosphorylated by CDK/CYC complex, activator-type MYB transcription factors (Act-MYBs; i.e., MYB3R1 and MYB3R4) trigger G2/M-specific gene expression [106,107], while repressor-type MYBs (Rep-MYBs; i.e., MYB3R1-dual function-, MYB3R3, and MYB3R5) maintain these genes repressed in post-mitotic cells and restrict their expression in proliferating cells [65]. In this scheme, under normal growth, Rep-MYBs upon CDK phosphorylation are degraded via the ubiquitin-proteasome system, while, in response to DNA damage, they accumulate to high levels. It appears that if the *Rep-MYBs* are not transcriptionally regulated in response to DNA damage, the SOG1-targeting of CKIs, resulting in the inhibition of CDK phosphorylation activity [108], might contribute to the stabilization of the Rep-MYBs, allowing them to ensure a high repression level of a subset of G2/M genes, thus, leading to the blockage of the cell cycle [86,109] (Figure 2).

While the key role in cell cycle arrest upon DNA damage of the Rep-MYBs occurs in a SOG1-dependant manner, the analysis of the Arabidopsis double mutant *wee1 sog1* revealed also the evidence of a SOG1-independent pathway in plant DDR regulation [87]. Recent global transcriptomic and mutant analyses [86,110,111,112] have strengthened this hint and pushed towards an implication of the E2F-RBR1 module in the plant DDR (Figure 2). As already mentioned, Rb-related proteins and their interactions with the E2F TFs are evolutionary conserved and regulate both G1/S and G2/M progression. As part of the DREAM complexes, they are also involved in stem cell maintenance, asymmetric division, and cell differentiation, as well as genome stability.

In addition, and as already described in the animal field [113,114], the implication of these cell cycle regulators in DDR has clearly emerged [60]. As expected, a transcriptional control of DDR genes dependant notably on RBR1, E2FA, as well as E2FC, has been observed in Arabidopsis [62,110,111,115]. Also, RBR1 and E2FA have been shown to be recruited directly at the sites of DNA lesions, where they co-localise with γ-H2AX foci in an ATM- and ATR-dependent manner, suggesting their close participation in repair mechanisms [110,111,116]. 

Similarly to its hub function in the DREAM complex, RBR1, in a CDKB1/CYCB1;1-dependent manner [111], could play the role of a platform recruiting at γ-H2AX foci different actors of the DNA repair machinery (Figure 2). For instance, RBR1 physically interacts with BRCA1, a DNA repair protein of DSB lesions [110] and co-localizes with the RAD51 protein, a recombinase involved in HR repair pathway [111], supporting at least for RBR1 a strong implication in the maintenance of genome integrity upon DNA stress conditions, by promoting cell survival and establishing localized repair complexes. 

Recently, another key player of the cell cycle, the ubiquitin E3 ligase F-Box protein FBL17 ([70] and reference therein) involved in cell cycle progression and endoreduplication has been linked to the DDR in Arabidopsis. Indeed, *FBL17* loss-of-function plants exhibit a constitutive activation of DDR genes in a SOG1-independent manner. Moreover, FBL17 is also recruited at γ-H2AX sites, but only in the presence of RBR1, suggesting a new implication of this F-box protein in the ubiquitylation of proteins involved in DNA-damage signalling or repair [112].

Another interesting interconnection shows that the DNA repair protein SNI1 (Suppressor of NPR1, Inducible), a subunit of the SMC5/6 complex (Structural Maintenance of Chromosome) able to promote HR repair pathway, can directly bind the transactivation domain of E2FA and E2FB to repress their transcriptional activities. SNI1, thus, constitutes another node of regulation between cell cycle checkpoint and DNA damage repair [117].

While the implication of those plant cell cycle regulators in DDR appears clearly established, further analyses will be required to elucidate at the molecular level how these components act in DDR and whether true DREAM complex are involved, to efficiently trigger cell cycle arrest and recruit appropriate DNA repair machinery.

### 4.3. WEE1 A Key Intra-S Checkpoint Protein 

As previously mentioned, apart from its key function in metazoan cell cycle progression, upon DNA stress conditions, the Wee1 kinase/Cdc25 phosphatase module also plays a key role in the ATR/Chk1 pathway, by targeting Thr14 and Tyr15 of CDKs to induce the arrest or the resumption of the cell cycle, respectively [41]. The model plant Arabidopsis lacks functional Cdc25 and Chk1 proteins [43], but ATR and WEE1 proteins have been both characterized and appear to trigger cell cycle arrest in a plant-specific manner [42,118]. 

Among the 108 identified ATM/ATR phosphorylated proteins in plants, only 69 proteins are orthologues with the ones of the mammal ATM/ATR phosphoproteome [119]. Interestingly, the blocking of the cell cycle progression is independent from the WEE1 phosphorylation of the Arabidopsis PSTAIRE-CDKA;1 but might depend on the phosphorylation of other substrates [45]. 

Thus far, the transcriptional induction of *WEE1* in an ATM/ATR-SOG1-dependent manner upon DNA damage stress conditions (zeocin, cadmium exposition, and root-nematode-infection) or replicative stress (HU, aphidicolin) is clearly observed and results in cell cycle arrests in G1/S or G2/M phases [44,85,120,121,122]. Nevertheless, until recently, how WEE1 is actually able to activate those cell cycle checkpoints was still a major question in plants.

**Figure 3:**
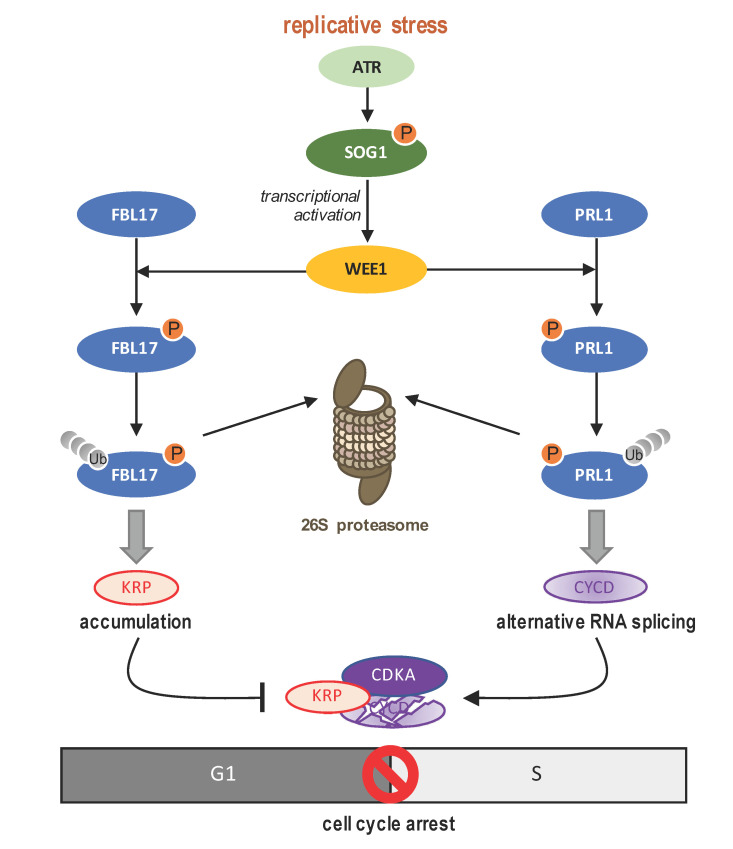
Upon replicative stress, WEE1 targets at least two substrates to efficiently establish a blockage in G1/S transition. As an important DDR player in plant, WEE1 is activated in an ATR/SOG1-dependant manner. If WEE1 implication during G2/M phase transition has been already described, recent works highlight its implication during the G1/S phase transition. 1—By phosphorylating the FBL17 E3 ubiquitin ligase, WEE1 triggers its proteasome degradation resulting in further accumulation of KRPs, which block the cell cycle progression [123]. FBL17 being also recruited at DNA lesion sites could target other regulators to fine tune DNA repair mechanisms [112]. 2—By targeting PRL1 a subunit of the MAC complex involved in RNA splicing, WEE1 promotes its selective degradation resulting in a non-functional MAC complex. As a consequence, *CYCD* mRNAs are not properly spliced compromising the activation of CDKAs and the cell cycle progression [124].

By identifying direct substrates of the WEE1 kinase, the Yan laboratory finally shed new light on the mechanistic action of the plant WEE1 kinase [123,124] to induce a specific DDR-dependent cell cycle arrest process (Figure 3). They demonstrated that, upon replicative stress, WEE1 interacts with and phosphorylates the F-box protein FBL17 inducing its ubiquitylation and further 26S proteasome degradation [123]. In line with its previously described role in KRP degradation [68,69,70,125,126], the loss of FBL17 proteins results in KRP accumulation causing CDK/CYC inhibition, and therefore a blockage in S phase progression [123]. As the p21 and p27 CDK inhibitors are targeted by the mammal Skp2 [127,128] and given that the human Wee1A is able to target Skp2 in vitro, even if these data need further explorations, it is tempting to believe that the mechanistic WEE1-FBL17/Skp2-CKIs-CDKs pathway might be conserved between plants and animals to trigger cell cycle arrest [123].

The second WEE1 substrate identified in response to replicative stress [124], is the PRL1 (PLEIOTROPIC REGULATORY LOCUS 1) WD40 protein, a core subunit of the Arabidopsis MAC (MOS4-associated Complex) complex, implicated in the control of RNA intron splicing and miRNA biogenesis [129]. It was shown that WEE1-phosphorylation of PRL1 leads to its ubiquitylation and subsequent proteasome degradation, consequently leading to the inhibition of the MAC complex, which directly affects the intron splicing of cell cycle genes among which are *CYCD1;1* and *CYCD1;3*. Thus, upon replicative stress, the intron retention on these *CYCDs* results in altered proteins unable to activate CDKA;1 leading to an arrest of cell cycle progression [124]. Similarly to the animal field [130], and given that among others, *SOG1, ATM* and *ATR,* are also subject to alternative splicing, these data further support the implication of alternative splicing in the plant DDR control (reviewed in [131]). 

Altogether, these new data clearly emphasize the complex role of plant WEE1 in DNA stress response: WEE1 controls cell cycle arrest by combining the stabilization of CKI proteins, together with the establishment of reduced CDK activities through alternative splicing.

### 4.4. Switch towards Endoreduplication: A Main Strategy in Plant DDR

Endoreduplication has been recognized for its implication in stress responses (reviewed in [103,132]), as it offers the benefits of avoiding proliferation of cells with damaged DNA and the occurrence of dying tissue, putative basis of altered organ development and/or a breach for pathogen infections, while providing a mechanism for cells to adapt metabolic and/or gene expression to combat the stress.

At a mechanistic level, the shift towards endoreduplication relies mostly on the repression of mitotic activities combined with the activation of endoreduplication onset. Upon DSB DNA damage, the blockage of the cell cycle in G2/M is promoted in an ATM/ATR-SOG1 dependent manner by the activation of distinct mechanisms resulting in the inhibition of mitotic CDK-CYC complex [86,102,133]. Accordingly, at both transcriptional and/or protein levels, CYCAs and mitotic CYCBs as well as CDKB1;1 and CDKB2;1 are down regulated. Simultaneously, positive regulators of endoreduplication onset, such as CDK suppressors, i.e., WEE1 and CCS52As, or the E2FA TF [23] are accumulating. 

Similarly, the induction of CKI regulators is to be noticed. Indeed, members of the plant CKI families, the SMR and KRP, have been largely characterized to block cell cycle progression through the negative regulation of CDK/CYC complexes and induction of the endocycle [28,48,108,134]. Despite this substantial functional redundancy, some SIM/SMRs (i.e., SIM, SMR1, SMR2, and SMR11 [28,135] and KRPs [51,52,53,124,136,137] are clearly dedicated to developmental and cell size control processes while others appear to exhibit a specialisation in stress response, and in particular to DNA damage stress. Among those latest, SMR4, SMR5, and SMR7 constitute key cell cycle checkpoints in response to DNA damage. Thus, in Arabidopsis upon DNA damage caused by HU treatment or cadmium exposure, *SMR4*, *SMR5,* and *SMR7* are transcriptionally activated, their promoter, as the one of *KRP6*, being directly targeted by SOG1 [85,86,138].

This dodge to escape cell death has been clearly established in Arabidopsis and also in other plant species, such as cucumber, pumpkin, or radish, especially after UV irradiation or zeocin treatment [139,140,141], revealing a conserved strategy, at least among some dicotyledons. Nevertheless, in some plants cell polyploidy is not a general feature and endoreduplication is not used for cell survival in response to DNA damage [142]. For instance, in rice (*Oryza sativa*), as in other monocots, a strict cell leaf organisation is observed compared with the heterogenous mosaic-like cell structure of the dicots. Thus, to avoid disorganized morphogenesis and development, and unlike in Arabidopsis where *CDKB2* expression is inhibited upon DSB [102], the accumulation of *Os*CDKB2 is favoured in rice and trigger cell differentiation and enhanced DNA damage repair capacity during cell cycle arrest [142]. 

Those data illustrate that DDR and checkpoint controls are also specialised among the plant kingdom [143] and it comes into views that they are still largely unexplored in crop plants.

## 5. Concluding Remarks

Even if plants can cope with high exposition to DNA-damage causing agents [144], maintenance of their genome integrity as for all living organisms is indispensable. It is clear that cell cycle progression and DNA damage responses are intimately linked to settle checkpoint controls and determine the fate of the damaged cells; nevertheless, the latest research data emphasize the complexity of the DNA stress responses. 

The plant DDR pathway has to adapt not only to the type of DNA lesion detected but also to the cell cycle phase as well as the specificity of the given tissue to trigger either endocycle or cell cycle arrest. In this objective and given their sessile lifestyle, it seems that plants have taken advantage of the huge number of cell cycle players to interfere in DNA damage stress response and fine tune cell cycle progression and organ development upon genotoxic stress conditions.

The most recent analyses have mainly focused on how plants respond to DNA damage occurrence; however, it is clear that this needs to be further explored in plant models such as Arabidopsis, as well as in crops to better valorise efficient DNA repair mechanisms. Following the repair of DNA lesions, the next step will be to understand how plants also actively terminate checkpoints to ensure the continuity of the organ growth revealing the amazing plasticity of plant development.
